# Lateral Force Microscopy of Interfacial Nanobubbles: Friction Reduction and Novel Frictional Behavior

**DOI:** 10.1038/s41598-018-21264-6

**Published:** 2018-02-15

**Authors:** Chih-Wen Yang, Kwan-tai Leung, Ren-Feng Ding, Hsien-Chen Ko, Yi-Hsien Lu, Chung-Kai Fang, Ing-Shouh Hwang

**Affiliations:** Institute of Physics, Academia Sinica, Nankang, Taipei 115, Taiwan

## Abstract

Atomic force microscopy is used to conduct single-asperity friction measurements at a water-graphite interface. Local mapping of the frictional force, which is based on the degree of the cantilever twisting, shows nearly friction-free when a tip scans over a nanobubble. Surprisingly, apart from being gapless, the associated friction loop exhibits a tilt in the cantilever twisting versus the tip’s lateral displacement with the slope depending on the loading force. The sign of the slope reverses at around zero loading force. In addition, the measured normal and lateral tip-sample interactions exhibit unison versus tip-sample separation. Theoretical analysis, based on the balance of forces on the tip originated from the capillary force of the nanobubble and the torsion of the cantilever, offers quantitative explanations for both the tilted friction loop and the unison of force curves. The analysis may well apply in a wider context to the lateral force characterization on cap-shaped fluid structures such as liquid droplets on a solid substrate. This study further points to a new direction for friction reduction between solids in a liquid medium.

## Introduction

Interfacial friction in ambient environment has been extensively studied due to its importance in fields such as micro- and nanoelectromechanical systems (MENS and NENS)^1,2^, shear in particle or colloidal suspensions^3,4^, froth flotation^5,6^, and micro-fluidics^7–11^. In practice, low-friction surfaces can be achieved chemically by coating the surface with lubricants such as silicone oil, glycerin, jelly-type materials, and other modified molecular thin films^2,12^. The interfaces where friction arises are typically heterogeneous at the nanometer scale. Single-asperity friction measurements based on contact-mode atomic force microscopy (AFM) provide tribological information at nanometer or sub-nanometer resolution, which can be directly related to surface topography, shedding new light on the microscopic origin of friction. This kind of measurements have been applied to study nanotribological properties of various solid surfaces under vacuum or ambient air environment^13–16^. However, studies under liquid environment as well as on nanostructures of fluid phases remain lacking. In this work, we present the lateral force microscopy of individual nanobubbles at an interface between water and highly oriented pyrolytic graphite (HOPG), a mildly hydrophobic substrate.

It has been found that the drag between water and hydrophobic solid walls is often reduced compared with the no-slip boundary condition typically seen between water and hydroplilic walls^7–9,17^. This phenomenon is known as boundary slip and its origin remains an important mystery in fluid dynamics. Recent studies suggest that gas nanobubble at the hydrophobic-water interfaces, known as interfacial nanobubbles (INBs), may be responsible for the high slippage of water at hydrophobic walls^17–23^. The INBs are nanometer-high, gas-containing and cap-shaped structures^21,23–30^ that are formed spontaneously at water-hydrophobic interfaces. They have been widely considered as gas bubbles in the nanometer scale. However, some experiments using surface force apparatus (SFA) or atomic force microscopy (AFM) have shown that the gas bubbles trapped at the solid surface acted as an anti-lubricant and resulted in high friction^10,11^. AFM using a hydrophobic colloidal probe to study hydrophobic substrates in water also showed that the presence of INBs produced both higher friction and stronger long-range attractive force compared to the case with almost no INBs^31^. Such observations have been attributed to larger capillary bridges caused by nanobubble coalescence. Despite efforts from a number of research groups, the role played by INBs in the phenomenon of boundary slip remains unclear^17–23^. Previous experimental studies used probes of tens of microns or larger in size, so microscopic details in measurements such as the number and dimension of INBs are not resolved. As we will show here, studying the tribological effect of individual INBs with single-asperity lateral force microscopy provides valuable information on the microscopic interactions between INBs and the tip, thereby helping understand the effects of INBs on friction and boundary slip.

## Results

In this work, the AFM topographic images were acquired with the contact mode. The preparation of HOPG surface with presence of INBs in water and AFM operation are described in the part of Methods. The normal bending of the cantilever (measured as the Y signal in the beam-deflection detection) was used as the feedback input to maintain a constant loading force during tip scanning across the sample surface. The friction between the sample and the tip during the lateral scanning produces twisting of the cantilever, which can be measured as the X signal in the beam-deflection detection. In this work, the X signal is often plotted in terms of the lateral force, a convention adopted in most studies of lateral (friction) force microscopy. A map of the X signal can therefore be acquired along with a topographic image and it is generally displayed as a lateral force map (or a frictional force map).

### Lateral Force Microscopy of Nanobubbles at a Water-HOPG Interface

**Figure 1:**
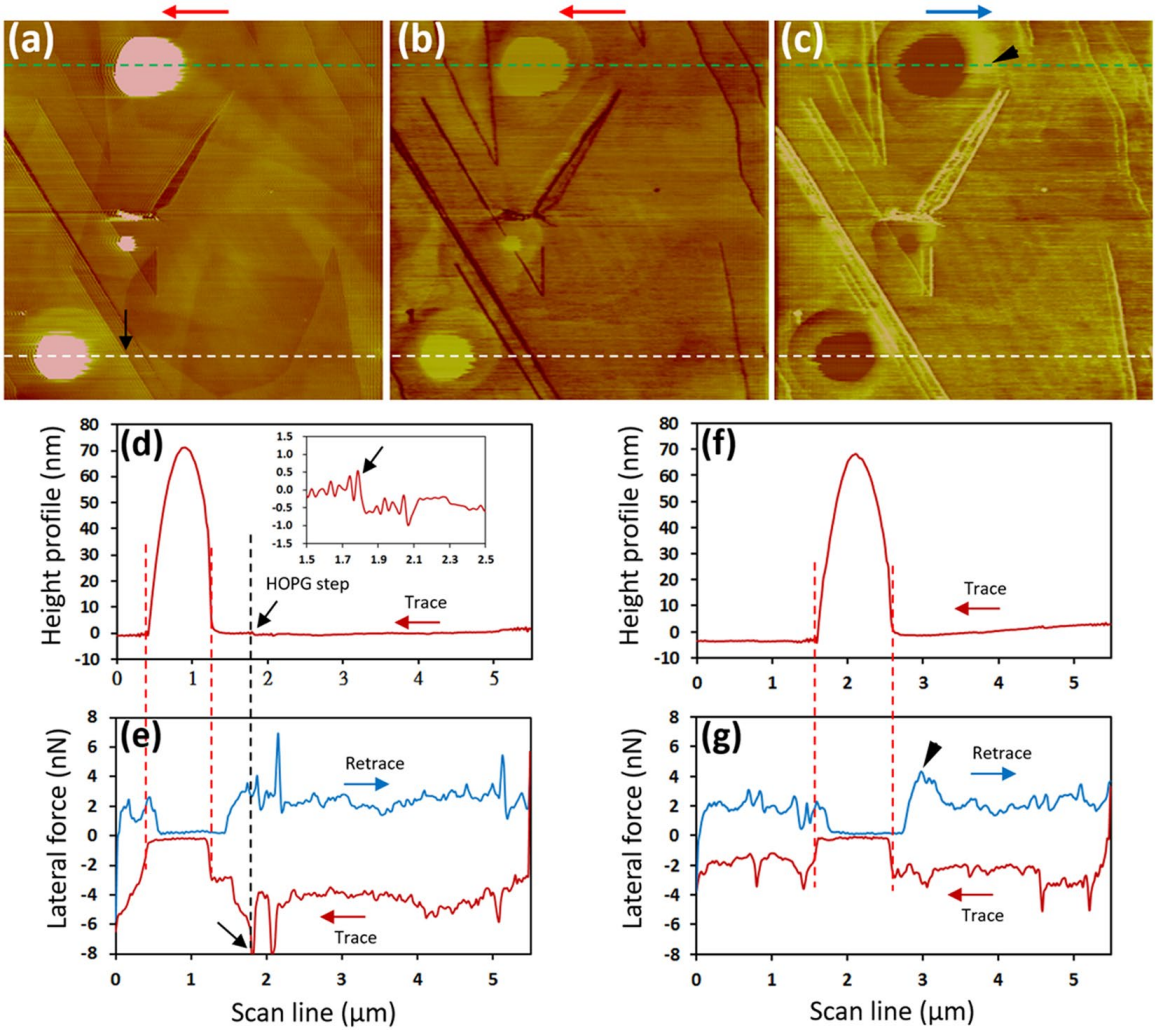
Lateral force microscopy of a HOPG/water interface with the presence of INBs acquired at a normal loading force of −50 pN and scan rate of 10 lines/s (110 μm/s). The height image in the trace scan (**a**) and the X-signal maps in a trace scan (**b**) and retrace scan (**c**) were acquired simultaneously. The trace and retrace scan directions are indicated with an arrow above each panel. The corresponding height profile in a trace scan (**d**) and the X-signal profile in the trace and retrace scans (**e**) were measured along the white-dashed lines marked in (**a**–**c**). Inset in (**d**) shows a magnified view near a HOPG step. The corresponding height profile in a trace scan (**f**) and the X-signal profile in the trace and retrace scans (**g**) were measured along the green dashed lines marked in (**a**–**c**). Normal spring constant, k_n_ ~ 0.08 N/m. The torsional spring constant was estimated as k_t_ ~ 16.0 N/m.

Figure 1a shows a height image of a HOPG-water interface acquired at an applied normal load of −50 pN and a scan rate of 10 lines/s (110 μm/s). Two large cap-shaped INBs and a small one were present. Figure 1b and c show the corresponding X-signal maps acquired in the trace and retrace directions, respectively; the INBs appear brighter (darker) than other regions in the trace (retrace) scan. Figure 1d displays the height profile in the trace scan across an INB along the white dashed line in Fig. 1a; Fig. 1e shows the corresponding X-signal acquired in the trace and retrace scans. Interestingly, the X signal nearly overlapped in traces and retrace scans when the tip scanned over the INB, whereas a clear gap was detected outside the INB, indicating much smaller friction over the INB than elsewhere. We note that the peaks in the lateral forces shown in Fig. 1e are due to the tip sliding across HOPG step edges. A black arrow indicates a step edge in the topographic image (Fig. 1a), height profile (Fig. 1d) and X-signal profile (Fig. 1e).

Figure 1f displays the height profile along the green dashed line across an INB on the top in Fig. 1a; Fig. 1g shows the corresponding X-signal acquired in the trace and retrace scans. Similarly, the X signal nearly overlapped when the tip scanned over the INB, but not outside the INB. Notice that a small bright region (indicated by a black arrowhead) is seen just to the right of the INB in Fig. 1c. In the retrace profile in Fig. 1g, there is also an increase in the lateral force when the tip scanned away from the INB (indicated by a black arrowhead), followed by a decrease to a stable value in the flat region. This increase in the lateral force cannot be explained by HOPG step edges because there is a region of ~2 μm to the right of the INB where no step edges are present (Fig. 1a). A small bright region to the right of an INB can also be seen for the INB near the bottom-left corner (Fig. 1c), but the effect is not as prominent due to the presence of step edges. The origin of this lateral force increase when a tip scans past an INB will be proposed in the discussion section later.

We also conducted lateral force microscopy of the same region at a reduced scan rate of 5 lines/s (55 μm/s), as shown in Supplementary Fig. S1. The hysteresis gap shown in Fig. S1e and g becomes smaller (hence smaller friction) over the region outside the INB when compared with Fig. 1e and g. Similarly, the hysteresis gap almost disappeared when the tip scanned over the INB, which is a characteristic we consistently detected in several other similar experiments.

### Effect of the Applied Normal Force on the Lateral Tip-Nanobubble Interaction

**Figure 2:**
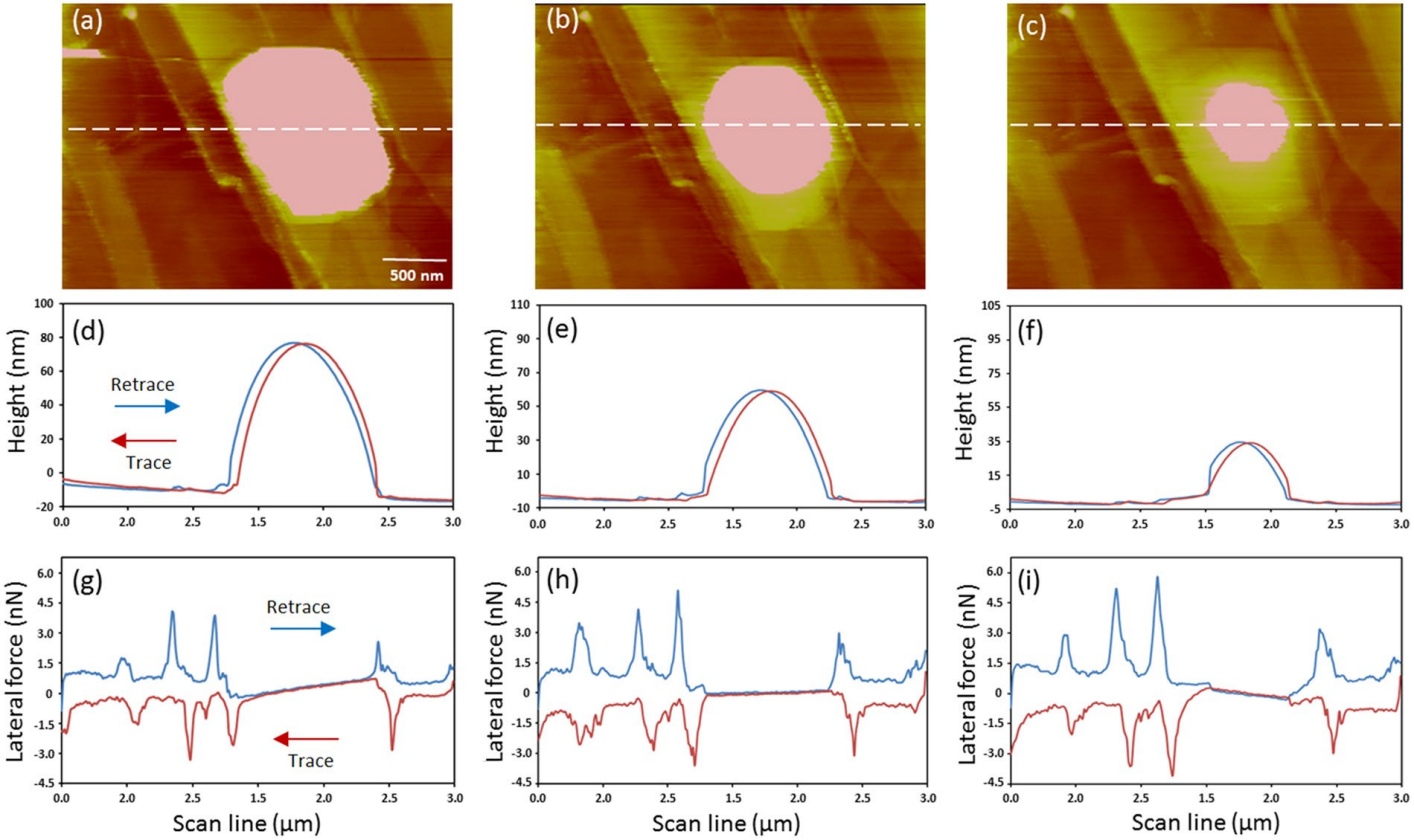
(**a**–**c**) Height images of an INB acquired at the normal force of −800, −90 and +650 pN, respectively. (**d**–**f**) Trace/retrace height profiles across the INB along the dashed line in (**a**–**c**), respectively. (**g**–**i**) Show the corresponding X-signal profiles along the dashed line in (**a**–**c**), respectively. Notice the pronounced positively and negatively tilted friction loops under the normal force of −800 and +650pN, respectively. Scan rate = 5 line/s (30 μm/s). Normal spring constant, k_n_ ~ 0.07 N/m. The torsional spring constant, k_t_, was estimated as ~14.2 N/m.

When the loading force was changed, an interesting phenomenon in the X-signal was detected while the tip slid over an INB. Figure 2a–c show the height images of an INB under the loading force of −800, −90, and +650 pN, respectively. As the normal force increased, the INB appeared smaller in both the lateral size and height. Figure 2d–f are the trace/retrace height profiles across the INB along the dashed line in Fig 2a–c, respectively. The corresponding X-signal profiles are shown in Fig 2g–i, respectively. Again, the overlap of the lateral force was seen when the tip scanned over the INB, regardless of the sign and magnitude of the loading force, indicating nearly friction-free on the INB. Note that the overlapped width decreased with increasing normal (loading) force, corresponding well to the decreasing apparent lateral size of the INB in the height image and height profile.

Surprisingly, the overlap regions of the X signal exhibited a tilt with a slope dependent on the loading force. The tilt was not evident in Fig. 2h as the loading force was set at −90 pN, similar to the measurement shown in Fig. 1e and g. When the loading force was set at a more negative value, −800 pN, the lateral force did not maintain a constant value but exhibited a noticeable tilt versus the lateral position of the INB (Fig. 2g) with roughly the same tilt slope for both the trace and retrace scans. When the normal force was set at a positive value, +650 pN, the sign of the tilt slope reversed (Fig. 2i).

We note that the measurements in Fig. 2 were reversible. The INB appeared larger again in the vertical and lateral sizes as the loading force was reduced, consistent with previous works^26,28^. Our extensive lateral force microscopy of the HOPG-water interface consistently indicated that the tilt in the lateral force over an INB exhibited a slope that varied with the loading force and changed sign at around zero setpoint force.

The tilting in the lateral-force loop has been observed previously and is attributed to the curved topography^32^ or lateral tilting of brush chains^33^. In those reports, no sign reversal in the tilt slope is detected, unlike here. In addition, in the current work, the tilt slope was seen only on INBs, but not on other areas of the surface. Previous explanations of the tilting in the lateral-force loop do not apply to our current observations. The mechanism for the tilting behavior on INBs, as shown in Fig. 2g–i, will be explained later.

### Force Curve Measurement on an INB

**Figure 3:**
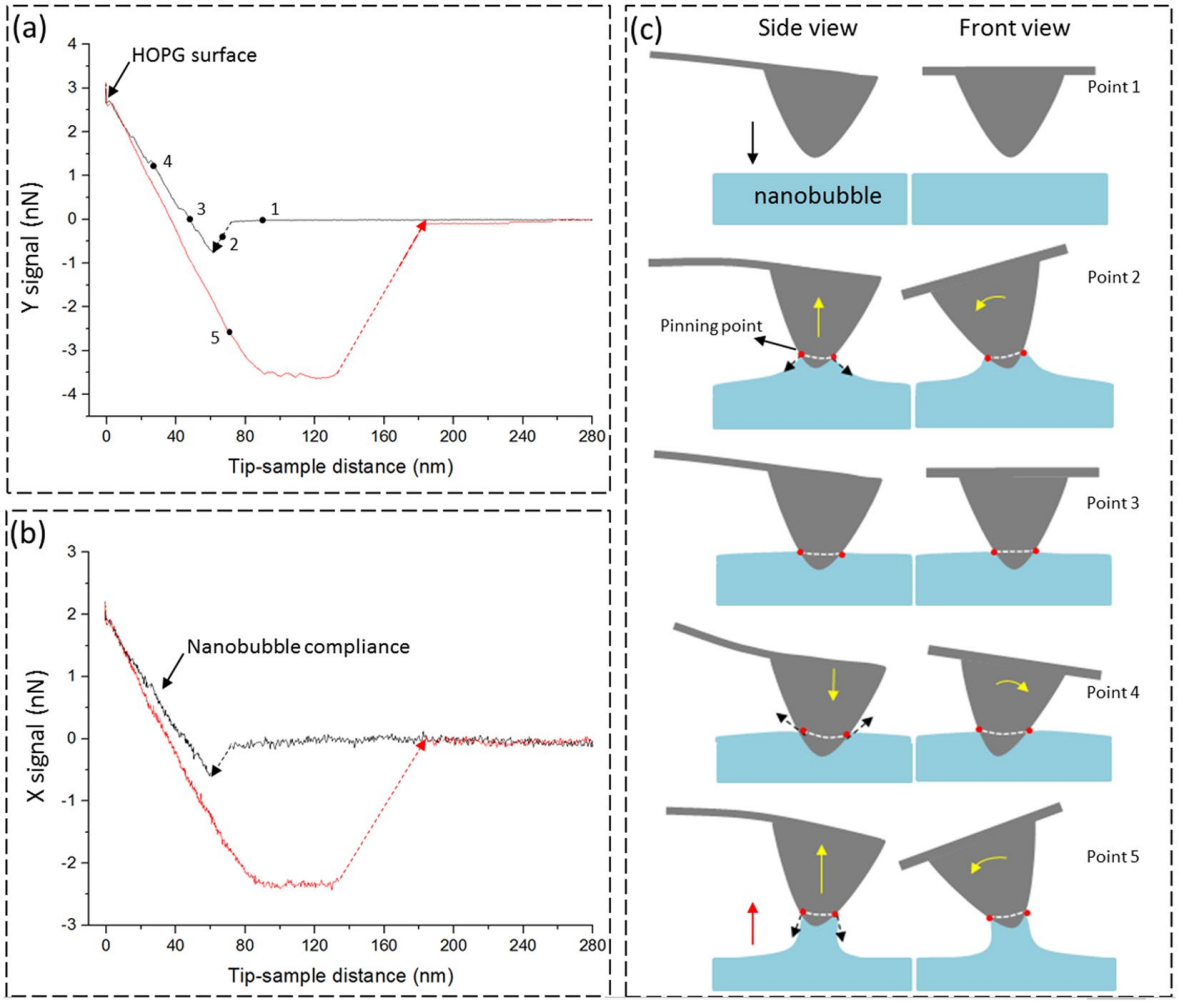
Approach-retraction force-distance curves measured on the INB shown in Fig. 2 and the corresponding schematics. (**a**) The Y signal (normal force); (**b**) the X signal. The ramp velocity for the piezo-scanner was 800 nm/s. The zero position was defined at the HOPG surface. (**c**) Schematic showing the tip and INB and the corresponding flexural bending (Y signal) and the twisting of the cantilever at different points indicated in (**a**). In point 1, the tip has not yet contacted the INB; in point 2, a snap-in occurs when the tip contacts the INB and a meniscus from the INB pulls the tip downward suddenly. There are flexural bending down and corresponding counterclockwise twisting of cantilever deflections in this state; in point 3, the tip further approaches the INB and the cantilever returns to the unforced condition. When the tip gets closer to INB as shown in point 4, the cantilever-tip induces both flexural bending up and clockwise twisting of deflections. During the retraction process as shown in point 5, the cantilever deflections changes to reflect flexural bending down and counterclockwise twisting due to the presence of the remaining capillary force.

To understand the interactions between an AFM tip and an INB, we measured the X and Y signals simultaneously by approaching an AFM tip toward an INB and then retracting away from the INB in the vertical direction. Figure 3a shows an approach-retraction curve of the normal force (Y signal) versus the tip-sample separation. In the approach curve, initially no force was detected. A snap-in, which pulled the tip toward the INB by a small distance suddenly, occurred when the tip touched the INB. Following the snap-in, a soft compliance region was detected when the tip penetrated deeper into the INB until the tip contacted the stiff HOPG substrate, when the slope of the force curves exhibited a sharp increase. When the tip was pulled away from the interface, a linear slope was detected in the initial stage of the retraction curve and the range was larger than the compliance region in the approach curve. The normal force became very negative until it reached a plateau at ~−3.5 nN. After the tip was pulled further back, snap-off occurred and the normal force changed suddenly to nearly zero. This behavior of the force curve is similar to those in previous measurements conducted on INBs^21,24,26,28,34,35^.

Amazingly, the X signal exhibited nearly identical trend as the Y signal during the approach-retraction cycle on the INB (Fig. 3b). The snap-in in the Y signal was also accompanied by a sudden change in the X signal, reflecting a sudden twist of the cantilever. Since there was no lateral scan during the measurement, the twisting of the cantilever had nothing to do with friction. While the X signal may be biased by misalignment of our measurement system in the beam-deflection detection, such a possibility was ruled out because the thermal spectra of the X and Y signals clearly exhibited different mechanical resonance characteristics: the X signal exhibited peaks at torsional resonance (TR) and lateral resonance (LR) frequencies^36^ and the Y signal exhibited peaks at vertical resonance (VR) frequencies (Supplementary Fig. S2).

Figure 3c illustrates the tip, INB, and the corresponding flexural bending and twisting of the cantilever at different points in the force curve measurements shown in Fig. 3a and b. It has been recognized that the snap-in of the approaching force curve (Fig. 3a) is caused by cantilever instability when the tip is brought close to the INB, when a capillary bridge is formed suddenly on the surface of the INB to pull the tip downward to reduce the surface energy of the capillary (point 2 in Fig. 3c)^34,35^. The roughly linear compliance region when the tip penetrated deeper into the INB suggests that the contact line of the capillary bridge on the tip surface was pinned by hydrophilic heterogeneities on the tip surface, which formed strong adhesion with water. The contact-line pinning of the capillary bridge on the tip surface might also cause sudden twisting of the cantilever during snap-in due to asymmetrical positions of the heterogeneities on the tip surface (Fig. 3c). While such a contact-line pinning may explain the force curve shown in Fig. 3b and the tilt of the lateral force (Fig. 2), it is a secondary effect as a more natural explanation exists without resorting to the presence of heterogeneities. We shall present next a theoretical analysis of the interaction between an AFM tip and an INB in which we derive the above synchrony of the force curves as well as the tilting of the profiles of lateral force during scanning over an INB.

### Theoretical Analysis

**Figure 4:**
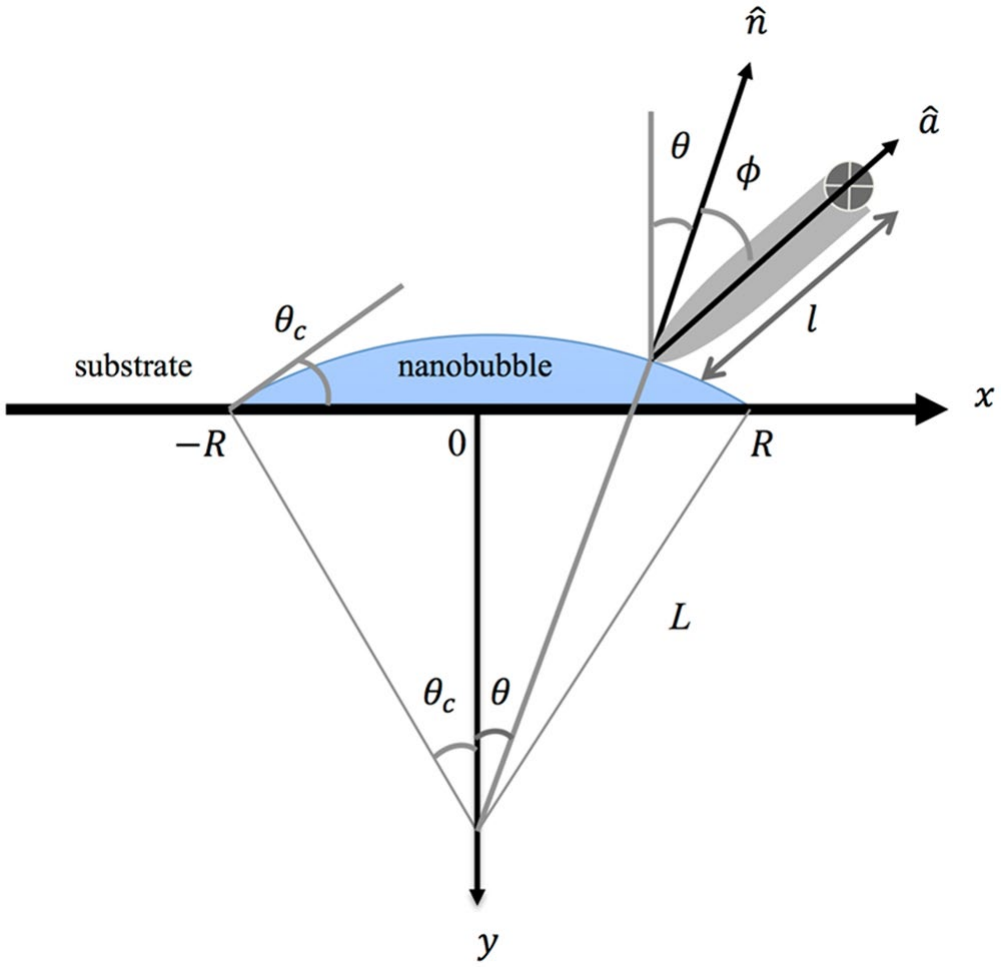
Coordinate system and angular variables associated with the nanobubble: shown are the radius of curvature $L$ of curved surface, bubble radius $R$, contact angle ${\theta }_{c}$, angular position $\theta$ on curved surface, unit normal vector $\hat{n}$, unit vector $\hat{a}$ along tip’s axis, tip’s angle of inclination $\varphi$, and distance $l$ between end of tip and pivot. Both $\theta$ and $\varphi$ are measured clockwise.

Consider a circular INB of radius *R* when viewed from above. Since INBs are mostly very flat^21,23–30^, we assume its upper surface takes the shape of a spherical cap of radius *L*. Define *θ*_*c*_ to be the interior contact angle between the substrate and the water-INB interface. Thus *L* = *R*/*sinθ*_*c*_. Generally, the flatness of the INB implies *L* ≫ *R*. Across the INB through its center, the position along the upper surface can conveniently be labeled by the angle *θ* that runs from -*θ*_*c*_ to *θ*_*c*_, as shown in Fig. 4. The unit normal vector $\hat{n}$ that points into the water is given by $\hat{n}=\hat{x}\sin {\theta }-\hat{y}\cos {\theta }$. To describe twisting, we introduce the unit vector $\hat{a}$ along the long axis of the tip, the angle *ϕ* between $\hat{a}$ and $\hat{n}$, and label the distance between the pivot and the end of the tip as $l$. These are all illustrated in Fig. 4.

When the INB is in contact with an AFM tip, the contact line forms a closed contour around the tip. Since the loading force in our experiment was relatively small, we assume the contact line stays within the hemi-spherical portion of the tip. In the absence of random pinning site, the contact line is symmetrical with respect to the local environment of the INB by virtue of $L\gg {r}_{T}$, where ${r}_{T}$ is the tip radius. The net capillary force applied on the tip by the INB points along $\hat{n}$ by symmetry, and so can be written as ${\mathop{F}\limits^{\rightharpoonup }}_{cap}={F}_{cap}\hat{n}$. For finite $\varphi$, ${\mathop{F}\limits^{\rightharpoonup }}_{cap}$ exerts a torque on the tip:

1 $${\mathop{\tau }\limits^{\rightharpoonup }}_{c{\rm{ap}}}=-l\hat{a}\times {\mathop{F}\limits^{\rightharpoonup }}_{cap}\,=l{F}_{cap}\,\sin \,\varphi \hat{z},$$

which is counter-balanced by the torsion of the cantilever

2 $${\mathop{\tau }\limits^{\rightharpoonup }}_{can}=-(\theta +\varphi )\mu \hat{z},$$

where $\mu$ is the torsional spring constant. Together, the total force and torque acting on the tip are given by

3 $$\mathop{F}\limits^{\rightharpoonup }={F}_{cap}\hat{n}-ky\hat{y}-k^{\prime} x\hat{x},$$

4 $$\mathop{\tau }\limits^{\rightharpoonup }=l{F}_{cap}\,\sin \,\varphi \hat{z}-(\theta +\varphi )\mu \hat{z},$$

where $k\text{'}$ is the lateral spring constant of the cantilever. Common slab shape of cantilever entails $k\text{'}\gg k$, so $x\,\,$is negligible and irrelevant. Since the restoring forces of bendings of the cantilever apply at the pivot, they exert no torque.

Consider the case of slow scanning such that the system always relaxes to a state of mechanical equilibrium. At each instance, each component of the net force and torque then vanish:

5 $${F}_{y}={F}_{cap}\,\cos \,\theta +ky=0,$$

6 $${\tau }_{z}=l{F}_{cap}\,\sin \,\varphi -(\theta +\varphi )\mu =0.$$

Equation (6) enables us to solve for $\varphi$ as a function of $\theta$. Since $-{\theta }_{c}\le \theta \le {\theta }_{c}$ and ${\theta }_{c}\ll 1$ for nanobubbles, we may do that iteratively. The first-order approximation is sufficient for our purpose:

7 $$\varphi (\theta )=\frac{\mu \theta }{l{F}_{cap}-\mu }+O({\theta }^{3}),$$

where only odd powers of $\theta$ appear by symmetry. The range of validity for $\varphi$ and stability of the equilibrium state depend on the value of $l{F}_{cap}$. It is straightforward to work that out. On the $\theta  > 0$ side:

- If $l{F}_{cap} > \mu  > 0:$ the solution has $\varphi  > 0$, $\frac{d{\tau }_{z}}{d\varphi } > 0$; it is unstable.
- If $0 < l{F}_{cap} < \mu :$ the solution has $\varphi  < -\theta  < 0$, $\frac{d{\tau }_{z}}{d\varphi } < 0$; it is stable.
- If $l{F}_{cap} < 0:$ the solution has $-\theta  < \varphi  < 0\,$, $\frac{d{\tau }_{z}}{d\varphi } < 0$; it is stable.

**Figure 5:**
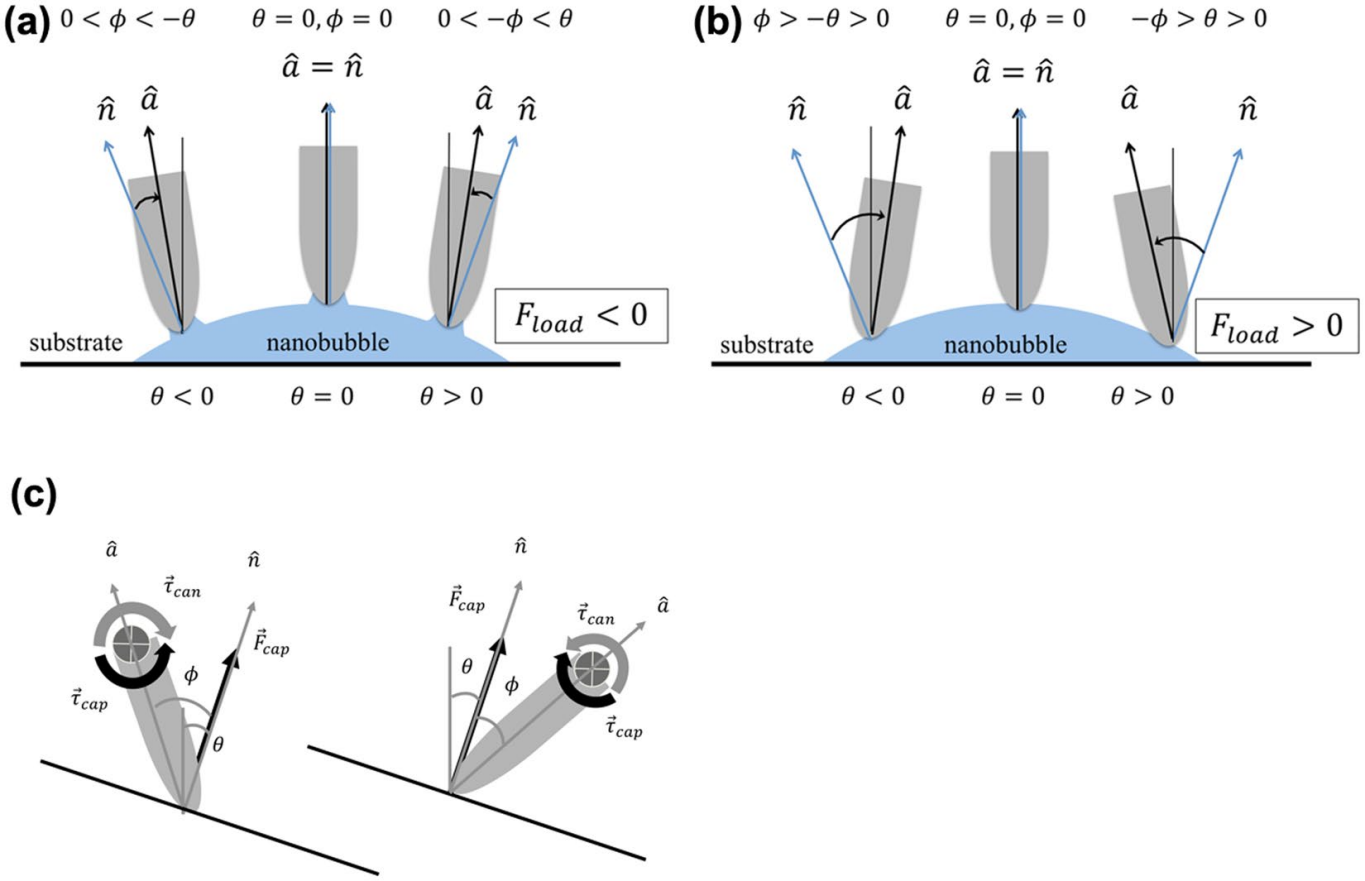
Theoretically determined stable configurations of an AFM tip in contact with a nanobubble for the case (**a**) when ${F}_{load} < 0$ (tip lifts the bubble), and (**b**) when ${F}_{load} > 0$ (tip presses onto bubble). The orientation of the tip is specified by the angle $\varphi$ of its symmetry axis. (**c**) illustrates the stability on the side of $\theta  > 0$ for $0 < l{F}_{cap} < \mu$ (left) and $l{F}_{cap} > \mu  > 0$ (right). While both configurations have zero net force and torque, when *ϕ* is increased infinitesimally by *dϕ* > 0, *dτ*_*z*_ < 0 on the left causes *dϕ* to diminish, whereas *dτ*_*z*_ > 0 on the right causes *dϕ* to grow.

Correspondences for $\theta  < 0$ can be obtained by the transformation $\{\varphi \to -\varphi ,\theta \to -\theta \}$. See Fig. 5 for an illustration.

To relate to our experiments, we deduce that $|l{F}_{cap}|$ is about an order of magnitude smaller than $\mu$ in our data, so only case (b) and (c) are relevant. From Eq. (5), the loading force is simply ${F}_{load}=-ky={F}_{cap}\,\cos \,\theta$, and the lateral force ${F}_{L}$ is the effective force that would generate the torque ${\mathop{\tau }\limits^{\rightharpoonup }}_{can}$. Hence, by combining Eqs (5) and (6), we find ${F}_{L}=\frac{(\theta +\varphi )\mu }{l}={F}_{load}\frac{\sin \,\varphi }{\cos \,\theta }\,.$

Substitution of $\varphi (\theta )$ from Eq. (7) into this equation yields

8 $${F}_{L}(\theta )\approx {F}_{load}\varphi (\theta )\approx \frac{\mu {F}_{load}}{l{F}_{load}-\mu }\theta +O({\theta }^{2}).$$

Finally, replacing $\theta$ by $x$ *via* $x={\rm{R}}\,\sin \,\theta /\,\sin \,{\theta }_{c}(\approx R\frac{\theta }{{\theta }_{c}}{\rm{to}}\,{\rm{first}}\,{\rm{order}})$ gives us the desired function ${F}_{L}(x)$:

9 $${F}_{L}(x)\approx \frac{\mu {F}_{load}{\theta }_{c}}{(l{F}_{load}-\mu )R}x+O({x}^{2}).$$

Since $|l{F}_{cap}| < \mu$, the slope and ${F}_{load}$ have opposite signs. This prediction is in complete agreement with our experimentally measured ${F}_{L}(x)$. Higher-order terms in Eq. (9) will render the profiles nonlinear, but they are too small to be detected experimentally due to small ${\theta }_{c}$.

Qualitatively, the above derivation shows that the torque that acts on the AFM tip by the capillary force of the INB undergoes a change of sign, as the tip moves along the curved surface of the INB. To maintain mechanical equilibrium, the cantilever swings accordingly, accompanied by also a sign change that manifests itself as a tilt in ${F}_{L}(x)$.

Although Eq. (9) is derived with the scanning experiment in mind, it applies equally to the case of vertical tip movement (where ${F}_{load}$ changes with $y$), provided that such movement is slow enough to maintain equilibrium. For $|l{F}_{load}|\ll \mu$ as is the case for our data, essentially ${F}_{L}(y)\approx -{F}_{load}(y){\theta }_{c}x/R$, i.e., the loading and lateral forces are in unison. $\theta (\text{hence}\,x)$ may be nonzero for a variety of reasons in experiment depicted in Fig. 3: The tip may have approached the bubble slightly off-center, or the bubble is not perfectly spherical to begin with. These situations should be quite common.

## Discussion

Recently, Tan *et al*. used an AFM tip to pull INBs along a lateral direction^37^. A neck was pulled out from an INB, which were observed with total internal reflection fluorescence microscopy. For a hydrophilic tip, the INBs survived the lateral pulling after their necks collapsed. The maximum pulling force was estimated as ~50 nN based on the change of the surface area of the capillary bridge imaged by optical microscopy and theoretical modeling. For a hydrophobic tip, the INBs were unpinned and slid across the substrate without breaking the capillary bridge and the maximum pulling force was estimated as ~100 nN. Figure 1g shows an increase in the lateral force of ~2 nN when a tip slid away from an INB, which may be the force to break the capillary bridge and is more than one order magnitudes smaller than the ~50 nN estimated by Tan *et al*. This indicates that our tips were more hydrophilic than the hydrophilic tips used by Tan *et al*.

The snap-in in the force curve measurements, as shown in Fig. 3a, also reflects the degree of hydrophobicity of the tip. It has been shown that a more hydrophobic tip experiences a larger snap-in and a larger snap-off force^35,38^. Our AFM tips were typically cleaned with UV or oxygen plasma treatment right before the experiments. They were relatively hydrophilic, as evidenced by the small snap-in shown in Fig. 3a. From our experience, an utterly hydrophilic tip experiences no snap-in, but such tips are very rare (<1% from our experiments). This indicates that most of the AFM tips retain a small degree of hydrophobicity even after the UV or oxygen plasma treatment. We speculate that a small part of the tip surface remains hydrophobic while most of the surface is hydrophilic. When the tip approaches and gets in contact with an INB, a capillary bridge forms connecting the INB and the hydrophobic area at the tip apex, initiating an attraction between INB and the tip. A snap-in occurs if the gradient of the attractive force is stronger than the spring constant of the cantilever. When the tip is pulled vertically away from the surface, the capillary bridge becomes thinner and eventually breaks, resulting in the snap-off force as shown in Fig. 3a. The hydrophobic area of the tip determines the cross section of the capillary bridge. A tip with a larger hydrophobic area (a more hydrophobic tip) thus experiences a larger snap-off force and the capillary bridge can be stretched more before breaking. This would explain the force curves measured with tips of different hydrophobicity.

**Figure 6:**
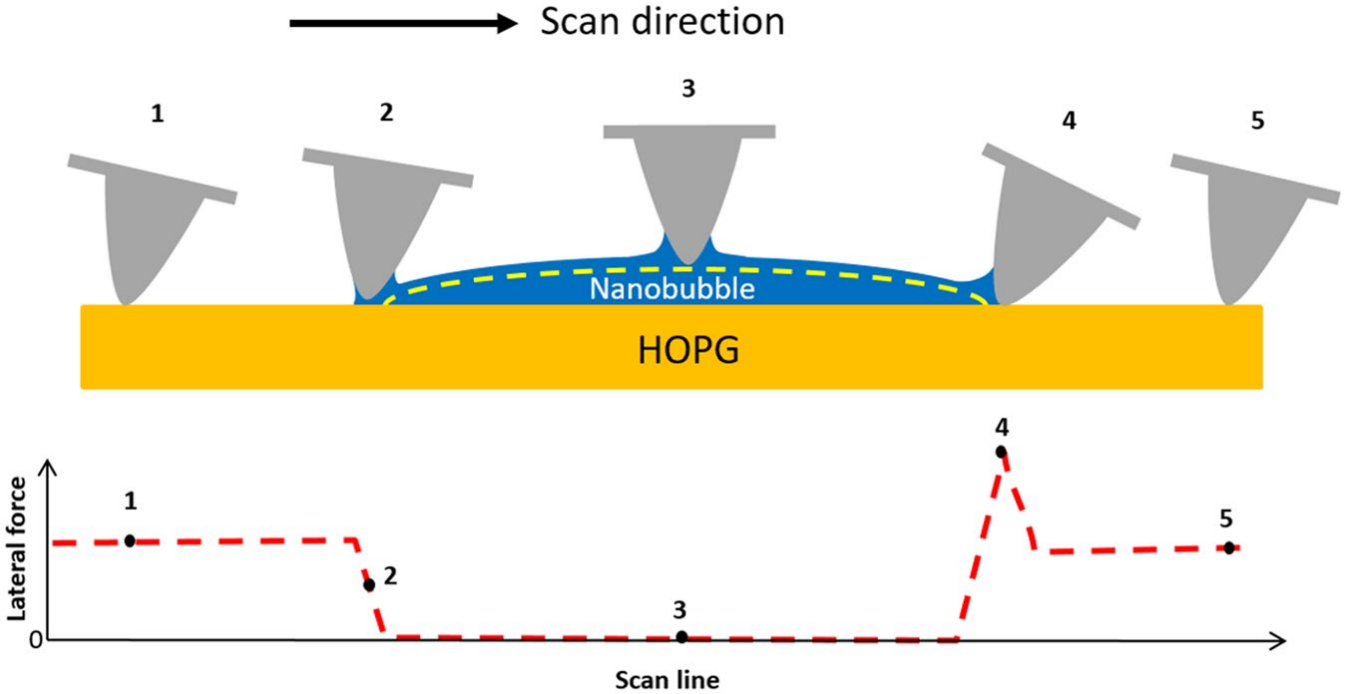
Schematic showing the lateral force experienced by an AFM tip when sliding across a hydrophobic surface with presence of an INB. The yellow dashed line indicates the profile of the INB traced by the scanning tip. The corresponding lateral force along the scan line is shown at the bottom. The medium (water) is not illustrated.

Figure 6 is a schematic illustration for the lateral force experienced by a scanning tip across an INB. When the tip slides on a hydrophobic solid in an area outside INBs (Point 1 in Fig. 6), it experiences a friction that produces a twisting of the cantilever. When the tip scans to an edge of an INB, friction starts to decrease. In addition, a capillary bridge forms and exerts a torques on the tip (Point 2). Both effects lead to the smaller twisting of the cantilever, which exhibits as a decrease in the lateral force. As the tip slides from the edge to the bulk of the INB, the friction reduces to a very small value because the tip no longer touches the hydrophobic solid under the condition of a constant loading force. The capillary force exerts a torques on the tip and produces a twisting of the cantilever, depending on the loading force as illustrated in Fig. 5. In Fig. 6, we simply choose the case with zero load (i.e. no twisting) to show no lateral force when the tip slides over the INB (Point 3). When the tip just slides past the INB, a capillary bridge connecting between the INB and the tip remains unbroken for some distance. The lateral force resulted from the elongated capillary bridge along with the friction from the solid substrate cause a strong twisting of the cantilever (Point 4). Twisting grows until the tip slides so far to the right that the capillary bridge breaks eventually, resulting in a peak of the lateral force indicated by a black arrowhead in Fig. 1g. This behavior is very similar to the snap-off in the force curve shown in Fig. 3a because their origins are the same. After the capillary bridge breaks (Point 5), the tip experiences only the friction from sliding on the solid, similar to the situation at Point 1, which is smaller than that at Point 4. The corresponding lateral force at different scan positions is also illustrated at the bottom panel of Fig. 6.

Because our tips were rather hydrophilic, the small hydrophobic area at the tip allowed formation of a small capillary bridge. Thus only a small increase in the lateral force (~2 nN) was detected when the tip slid past an INB (Fig. 1g). We expect a larger increase in the lateral force if a more hydrophobic tip is used. In some of our similar lateral force microscopy experiments, the AFM scan on INBs was sometimes not stable when the AFM tip was considerably more hydrophobic. This is probably because the capillary bridges formed between a more hydrophobic tip and INBs produce a larger contact area on tip apex, resulting in stronger break-off forces and relatively unstable AFM imaging. The bridges may even not break during the scan, pulling INBs away from their original sites, similar to the observations by Tan *et al*. using a hydrophobic tip. This also explains why a previous AFM work using a hydrophobic colloidal probe on hydrophobic substrates shows both high friction and strong long-range attractive force^31^.

The theoretical analysis asserts that the twisting of the cantilever is determined at equilibrium by the balance between the capillary force applied on the tip and the torsion of the cantilever. In principle, this analysis should also be applicable to lateral force microscopy on other cap-shaped fluid structures, such as liquid droplets, on a solid substrate, as long as the scanning is so slow that the viscous drag of the fluid is negligible. Notice that for droplets with larger contact angle than that of INBs, *θ* ≪ 1 is no longer valid and the full angular dependence needs to be maintained throughout. Interestingly, the assumption of mechanical equilibrium can be applied to the measurements up to the relatively high scan speed of 30 μm/s (Fig. 2), suggesting that INBs may have such a low viscosity that the drag it exerts on the sliding tip can be ignored. The low viscosity of INBs may also explain the phenomenon of boundary slip at a hydrophobic-water interface^18,39^. Our experiments still cannot determine whether the viscosity of INBs is smaller than that of water, but evidently INBs do not act as an anti-lubricant. Previous SFA and AFM works show that gas bubbles trapped at the solid surface act as an anti-lubricant and result in high friction. That may be due to INBs having a different nature from gas bubbles, as Hwang *et al*.^39–42^ proposed and explained that INBs are gas condensates with a low viscosity and low interfacial tension with water. The different nature between INBs and typical gas bubbles (semispherical and micron size or larger) is also supported by the fact that stable AFM imaging of INBs can be achieved easily but stable AFM imaging of typical gas bubbles is never possible. However, further experimental and theoretical studies are needed to clarify this point. Nevertheless, this study indicates that INBs can be used as boundary lubricants between two solids in water, if one solid is rendered hydrophobic to promote formation of INBs and the other rendered hydrophilic to suppress formation of capillary bridges with the INBs. Recently, it has been reported that INBs also form in several liquids other than water^43^. As INBs contain only gas molecules, properly utilizing INBs may lead to environmental-friendly techniques for lubrication in liquid.

## Methods

### Sample Preparation

HOPG samples (lateral sizes of 12 mm × 12 mm, ZYB; Momentive) were freshly cleaved with scotch tape prior to each AFM experiment. All water was purified using a Milli-Q system (Millipore Corp., Boston) with a resistivity of 18.2 MΩ·cm. The solvent exchange process was used to produce nanobubbles at the HOPG-water interface^25–27^. Pure water was first injected into the fluid cell tip holder, then ethanol (99.9% from J. T. Baker) was injected to replace water, and finally pure water was injected again to replace ethanol. All the experiments were carried out at room temperature.

### AFM Experiments

AFM topographic images and lateral force images were simultaneously acquired with the contact mode using a commercial beam-deflection AFM (Bruker AXS MultiMode NanoScope V), which was equipped with a commercial open fluid cell. To enhance the force sensitivity for contact-mode imaging and force-distance measurements, a type of compliant silicon cantilevers with a Au-coated tip (MikroMasch, CSC38_A/Cr-Au, k = 0.01~0.08 N/m, nominal tip radius ~50 nm) was used. Before AFM scanning, the AFM tip was cleaned with UV treatment and the fluid cell was rinsed with ethanol several times. For acquiring a topographic image, the normal bending of the cantilever (measured as the Y signal in the beam-deflection detection) was used as the feedback input to maintain a constant normal force during tip scanning on the sample surface. For measurement of lateral forces (or frictional forces), the sample was scanned laterally in a direction perpendicular to the long axis of the AFM cantilever. The friction between the sample and the tip during the lateral scanning produces twisting of the cantilever, which can be measured as the X signal in the beam-deflection detection. In this work, the X signal is often plotted in terms of the lateral force, a convention adopted in most studies of lateral (friction) force microscopy. A map of the X signal can therefore be acquired along with a topographic image and it is generally displayed as a lateral force map (or a frictional force map). It has been shown that the changes both in the material property and topography (or slope) can contribute to the X signal. Analysis of the forward (trace) and backward (retrace) scans allows a distinction between these two effects^44^.

In determining the flexural and torsional spring constants of a cantilever, we followed the procedures published in the literature^45^. The normal and lateral forces can be calculated accordingly. In lateral (friction) force microscopy, a friction loop is acquired by recording the lateral forces in the trace and retrace scans along the same scan line at a constant loading force. The difference between the lateral forces in the opposite scan directions (i.e., the extent of the hysteresis) at a given sample position is considered to be twice of the friction force exerted at that point. A larger hysteresis loop indicates a stronger frictional force, and vice versa.

In previous AFM studies on INBs, several imaging modes, including tapping, frequency-modulation, and PeakForce modes, were employed^21,23–30,39–42^. It was generally conceived that INBs could hardly be imaged with the contact mode due to the large normal and lateral forces associated with this imaging mode. In this work, the tip was first brought in contact with the solid-water interface at a positive set force to acquire a topography image. Then a sequence of scanning was performed at reduced set forces by small steps until the force became negative. We thus could reliably measure the topography and the corresponding lateral forces on INBs simultaneously over a range of set force.

## Electronic supplementary material

Supplementary Information

## References

[1] Bhushan B (2007). Nanotribology and nanomechanics of MEMS/NEMS and BioMEMS/BioNEMS materials and devices. Microelectronic Engineering.

[2] Liu H, Bhushan B (2003). Nanotribological characterization of molecularly thick lubricant films for applications to MEMS/NEMS by AFM. Ultramicroscopy.

[3] Arranaga AB (1970). Friction reduction characteristics of fibrous and colloidal substances. Nature.

[4] Smith MI (2015). Fracture of jammed colloidal suspensions. Sci. Rep..

[5] Li Y, Zhu T, Liu Y, Tian Y, Wang H (2012). Imaging study of surfactant effect on bubble behavior in froth flotation. Water Sci Technol..

[6] Calgaroto S, Wilberg KQ, Rubio J (2014). On the nanobubbles interfacial properties and future applications in flotation. Minerals Engineering.

[7] Tretheway DC, Meinhart CD (2002). Apparent fluid slip at hydrophobic microchannel walls. Phys. Fluids.

[8] Ou J, Rothstein JP (2005). Direct velocity measurements of the flow past drag-reducing ultrahydrophobic surfaces. Phys. Fluids.

[9] Ou RJ, Perot B, Rothstein JP (2004). Laminar drag reduction in microchannels using ultrahydrophobic surfaces. Phys. Fluids.

[10] Steinberger A, Cottin-Bizonne C, Kleimann P, Charlaix E (2007). High friction on a bubble mattress. Nature Materials.

[11] Maalil A, Bhushan B (2013). Nanobubbles and their role in slip and drag. J. Phys.: Condens. Matter.

[12] Misra R, Li J, Cannon GC, Morgan SE (2006). Nanoscale reduction in surface friction of polymer surfaces modified with Sc3 hydrophobin from Schizophyllum commune. Biomacromolecules.

[13] Jinesh KB, Frenken JWM (2006). Capillary condensation in stomic scale friction: how water acts like a glue. Phys. Rev. Lett..

[14] Liithi R, Meyer E, Howald L, Bammerlin M, Giintherodt H-J (1995). Friction force microscopy in ultrahigh vacuum an atomic-scale study on KBr (001). Tribology Letters.

[15] Meyer E (1992). Friction and wear of Langmuir-Blodgett films observed by friction force microscopy. Phys. Rev. Lett..

[16] Bhushan B (1999). Nanoscale tribophysics and tribomechanics. Wear.

[17] Ushida A, Hasegawa T, Nakajima T, Uchiyama H, Narumi T (2012). Drag reduction effect of nanobubble mixture flows through micro-orifices. Experimental Thermal and Fluid Science.

[18] Vinogradova OI (1995). Drainage of a thin liquid film confined between hydrophobic surfaces. Langmuir.

[19] De Gennes PG (2002). On Fluid/Wall Slippage. Langmuir.

[20] Lauga E, Howard AS (2003). Effective slip in pressure-driven Stokes flow. Journal of Fluid Mechanics.

[21] Lauga E, Brenner MP (2004). Dynamic mechanisms for apparent slip on hydrophobic surfaces. Phys. Rev. E.

[22] Wang Y, Bhushan B (2010). Boundary slip and nanobubble study in micro/nanofluidics using atomic force microscopy. Soft Matter.

[23] Li D, Jing D, Pan Y, Bhushan B, Zhao X (2016). Study of the relationship between boundary slip and nanobubbles on a smooth hydrophobic surface. Langmuir.

[24] Ishida N, Inoue T, Miyahara M, Higashitani K (2000). Nano bubbles on a hydrophobic surface in water observed by Tapping-mode atomic force microscopy. Langmuir.

[25] Lou ST (2000). Nanobubbles on solid surface imaged by atomic force microscopy. J. Vac. Sci. Technol. B.

[26] Zhang XH, Maeda N, Craig VSJ (2006). Physical properties of nanobubbles on hydrophobic surfaces in water and aqueous solutions. Langmuir.

[27] Zhang XH (2007). Detection of novel gaseous states at the highly oriented pyrolytic graphite/water interface. Langmuir.

[28] Yang C-W, Lu Y-H, Hwang I-S (2013). Imaging surface nanobubbles at graphite–water interfaces with different atomic force microscopy modes. J. Phys.: Condens. Matter.

[29] Seddon JRT, Lohse D (2011). Nanobubbles and micropancakes: gaseous domains on immersed substrates. J. Phys.: Condens. Matter.

[30] Lohse D, Zhang XH (2015). Surface nanobubbles and nanodroplets. Rev. Mod. Phys..

[31] Hampton MA, Donose BC, Taran E, Nguyen AV (2009). Effect of nanobubbles on friction forces between hydrophobic surfaces in water. Journal of Colloid and Interface Science.

[32] Egberts P, Han GH, Liu XZ (2014). Charlie Johnson, A. T., Carpick, R. W. Frictional behavior of atomically thin sheets: hexagonal-shaped graphene islands grown on copper by chemical vapor deposition. ACS Nano.

[33] Li A, Ramakrishna SN, Nalam PC, Benetti EM, Spencer ND (2014). Stratified polymer grafts: synthesis and characterization of layered ‘Brush’ and ‘Gel’ structures. Adv. Mater. Interfaces.

[34] Holmberg M, Kühle A, Garnæs J, Mørch KA, Boisen A (2003). Nanobubble trouble on gold surfaces. Langmuir.

[35] Walczyk W, Schönherr H (2014). Characterization of the interaction between AFM tips and surface nanobubbles. Langmuir.

[36] Ding R-F, Yang C-W, Huang K-Y, Hwang I-S (2016). High-sensitivity imaging with lateral resonance mode atomic force microscopy. Nanoscale.

[37] Tan BH, An H, Ohl C-D (2017). Resolving the pinning force of nanobubbles with optical microscopy. Phys. Rev. Lett..

[38] Song Y (2014). The origin of the snap-in in the force curve between AFM probe and the watergas interface of nanobubbles. Chem Phys Chem.

[39] Fang C-K, Ko H-C, Yang C-W, Lu Y-H, Hwang I-S (2016). Nucleation processes of nanobubbles at a solid/water interface. Sci. Rep..

[40] Hwang I-S, Yang C-W, Lu Y-H (2012). Evidence of epitaxial growth of molecular layers of dissolved gas at a hydrophobic/water interface. arXiv.

[41] Yang C-W, Lu Y-H, Hwang I-S (2013). Condensation of dissolved gas molecules at a hydraphobic/water interface. Chin. J. Phys..

[42] Lu Y-H, Yang C-W, Fang C-K, Ko H-C, Hwang I-S (2014). Interface-Induced Ordering of Gas molecules confined in a small space. Sci. Rep..

[43] An H, Liu G, Atkin R, Craig VSJ (2015). Surface nanobubbles in nonaqueous media: looking for nanobubbles in DMSO, formamide, propylene carbonate, ethylammonium nitrate, and propylammonium nitrate. ACS Nano.

[44] Bhushan, B. *Handbook of Nanotechnology*, Chap 17, Berlin, Springer (2004).

[45] Ortiz-Young D, Chiu H-C, Kim S, Voitchovsky K, Riedo E (2013). The interplay between apparent viscosity and wettability in nanoconfined water. Nature Communications.

