# Supplementary material for: Lateral Force Microscopy of Interfacial Nanobubbles: Friction Reduction and Novel Frictional Behavior
Source: Sci Rep. 2018 Feb 15;8:3125. doi: 10.1038/s41598-018-21264-6 (PMC5814448; doi:10.1038/s41598-018-21264-6)
Supplement: Supplementary file 1 — Supplementary Information [file 41598_2018_21264_MOESM1_ESM.pdf]

Supplementary Information

Lateral Force Microscopy of Interfacial Nanobubbles: Friction Reduction and Novel Frictional Behavior

Chih-Wen Yang, Kwan-tai Leung*, Ren-Feng Ding, Hsien-Chen Ko, Yi-Hsien Lu, Chung-Kai Fang, and Ing-Shouh Hwang*

Institute of Physics, Academia Sinica, Nankang, Taipei 115, Taiwan

*ishwang@phys.sinica.edu.tw; leungkt@ phys.sinica.edu.tw

Contents:

1. Supplementary Figures

Supplementary Figures

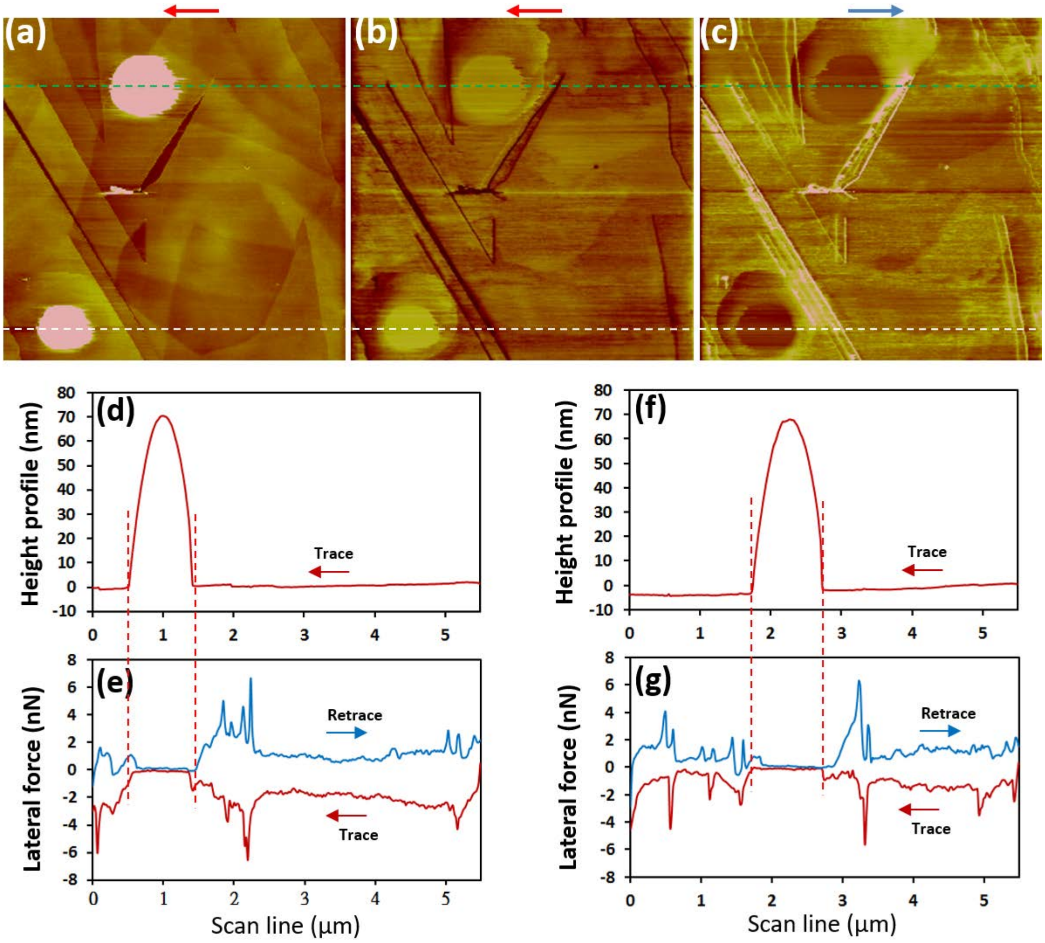

Figure S1. Lateral force microscopy of a HOPG/water interface with the presence of INBs at a normal loading force of -50 pN and scan rate of 5 lines/s (55 μm/s). The height image in the trace scan (a) and the X-signal maps in a trace scan (b) and retrace scan (c) were acquired simultaneously. The trace and retrace scan directions are indicated with an arrow above each panel. The corresponding height profile in a trace scan (d) and the X-signal profile in the trace and retrace scans (e) were measured along the white-dashed lines marked in (a)-(c). The corresponding height profile in a trace scan (f) and the X-signal profile in the trace and retrace scans (g) were measured along the green dashed lines marked in (a)-(c). Normal spring constant, k_n~ 0.08 N/m. The torsional spring constant is estimated as k_t~ 16.0 N/m.

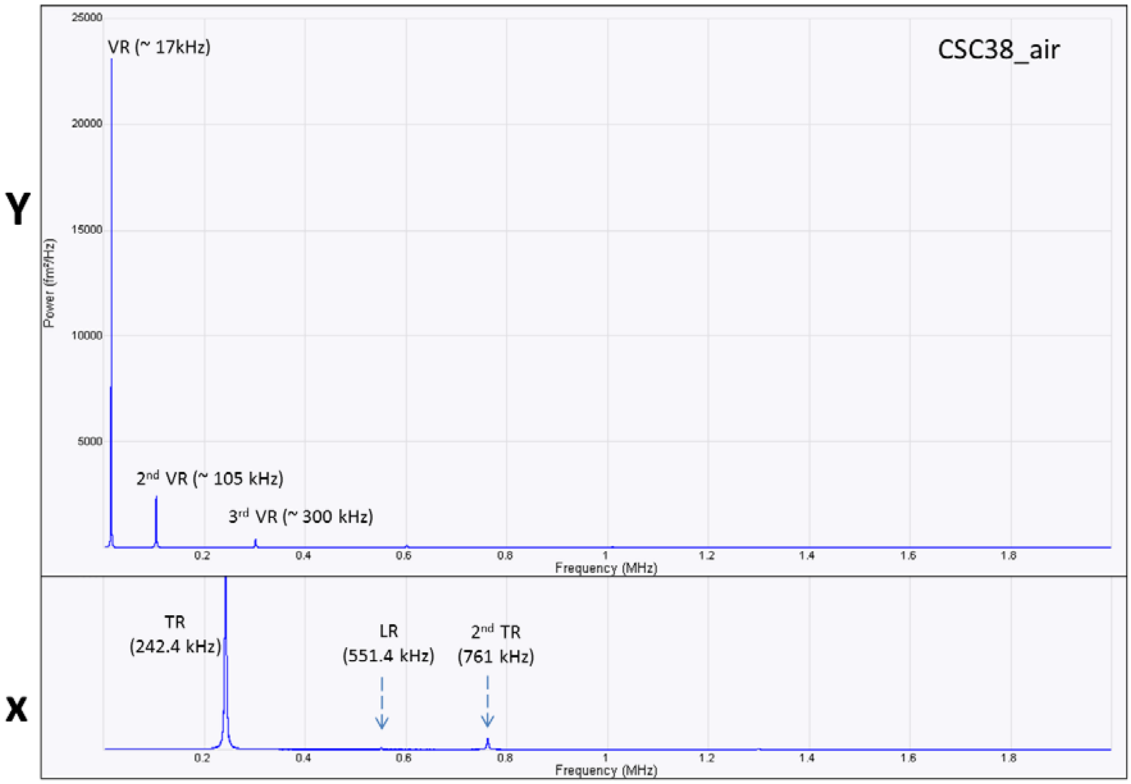

Figure S2. Thermal spectra of the X and Y signals of the cantilever.
